# Effects of a caregiver-inclusive assistive technology intervention: a randomized controlled trial

**DOI:** 10.1186/s12877-018-0783-6

**Published:** 2018-04-18

**Authors:** W. Ben Mortenson, Louise Demers, Marcus J. Fuhrer, Jeffrey W. Jutai, Jessica Bilkey, Michelle Plante, Frank DeRuyter

**Affiliations:** 1 Department of Occupational Science and Occupational Therapy, University of British Columbia, Vancouver, BC Canada; 2 GF Strong Rehabilitation Research Program, Vancouver, BC Canada; 3 International Collaboration on Repair Discoveries, Vancouver, BC Canada; 4 Centre de recherche de l’Institut universitaire de gériatrie de Montréal, Centre intégré universitaire de santé et de services sociaux du Centre-Sud-de-l’Île-de-Montréal, Montréal, PQ Canada; 5 École de réadaptation, Université de Montréal, Montréal, PQ Canada; 6 Eunice Kennedy Shriver National Institute of Child Health and Human Development, National Institutes of Health, Bethesda, MD USA; 7 Interdisciplinary School of Health Sciences, University of Ottawa, and Bruyère Research Institute, Ottawa, ON Canada; 8 Department of Surgery/Speech Pathology and Audiology, Duke University, Durham, NC USA

**Keywords:** Family caregivers, Older adults, Assistive technology, Randomized controlled trial

## Abstract

**Background:**

The principal aim of this study was to investigate whether a caregiver-inclusive assistive technology intervention improved older care recipients’ functional autonomy and decreased the perceived burden of their family caregivers compared to customary care.

**Methods:**

The study was a single-blind, mixed-methods, randomized controlled trial with baseline data collection and follow-ups at 6-, 22-, and 58-weeks after baseline evaluation, which was prospectively registered (ClinicalTrials.gov Identifier: NCT01640470. Registered 11/21/2011). Dyads comprising a care recipient and family caregiver were randomly assigned to either a caregiver-inclusive experimental group (*N* = 44) or a customary-care comparison group (*N* = 46). Eligible care recipients were aged ≥55 years and had one or more limitations with mobility or daily activities, and family caregivers provided at least four hours per week of assistance. Outcome measures were administered to both groups at baseline and at the three follow-up time points. The data collectors were blinded regarding participants’ intervention group. The primary outcome measures were the Functional Autonomy Measurement System to assess care recipients’ functional performance, and the Caregiver Assistive Technology Outcome Measure to assess caregivers’ burden. Qualitative interviews examined participants’ perceptions of the caregiver-inclusive and customary care interventions.

**Results:**

The experimental intervention addressed significantly more dyad-identified problematic activities, but caregiver involvement was evident in both groups and outcomes were not significantly different over time. In both groups, care recipients’ functional autonomy declined significantly (*P* < .01), and caregivers’ activity-specific and overall burden decreased significantly (*P* < .01).

**Conclusions:**

Given the unintended congruence between the caregiver-inclusive and customary care interventions, the overall findings lend support for the provision of assistive technology to reduce caregiver burden.

## Background

National population projections suggest that by 2030, one in five Americans will be 65 years and older, and the population of older adults will continue to grow into the year 2060 [1]. While disability rates appear to be stabilizing, the prevalence of older adults with disabilities will increase substantially [2]. As people age they tend to experience increased physical, sensory, and cognitive limitations. For example, 50.7% of Americans over age 75 years reported in 2013 having one or more disabilities compared to 25.8% age 65-74 years [3].

Many individuals with disabilities rely on assistive technology, human assistance, or a combination of both to carry out their daily activities. Assistive technology (AT) refers to “devices and systems that include[…] both commercially developed or purpose-built devices that are designed to assist with specific tasks” [4]. Devices used frequently by older adults include wheelchairs, scooters, canes, walkers, grab bars, and bath seats [5]. As individuals age, their AT usage increases [6]. Three randomized controlled trials have revealed that using AT can reduce the decline of aging individuals’ self-reported performance in basic and instrumental activities [7, 8], and increase their self-efficacy and greater use of adaptive strategies [9].

People with disabilities also may receive assistance from formal and/or family caregivers. For example, 14.3% of adult Americans provided care to older family members or friends with chronic conditions, disabilities, or problems related to aging in 2014 [10]. Family caregivers (sometimes called informal caregivers) are the spouses, children, friends, and neighbors who provide emotional support and assistance with basic and instrumental activities of daily living, e.g., bathing, meal preparation, managing finances, and laundry [11]. On average, considering all of the care received, people with disabilities who receive caregiving assistance obtain 80% of that support from family caregivers, whereas the remaining 20% is provided by formal caregivers [12]. Only an estimated 2-3% of those who receive home care are without a family caregiver [13]. The annual value of family caregiving in the United States has been estimated at $470 billion [14]. Caregiving demands can become a burden which is emotionally and physically taxing [15]. Buhse [16] describes caregiver burden as “…a multidimensional response to physical, psychological, emotional, social, and financial stressors associated with the caregiving experience.” Because of the amount of unfunded assistance provided by family caregivers, burden, which leads to caregiver burn-out, poses a significant threat to the health care system.

Two systematic reviews have been conducted to understand the effects of AT provision on family caregivers [17, 18]. Both found that the provision of AT is generally associated with improved caregiver outcomes; e.g., when individuals with disabilities use AT, caregivers expend less physical and emotional energy. At the same time, the small number of experimental studies supporting this relationship was noted.

A recent, exploratory randomized controlled trial (RCT) addressed how deliberate inclusion of caregivers during the process of AT provision affects both care recipients (the AT users) and their family caregivers [19]. The findings indicated that AT increased users’ independence and satisfaction performing activities, and decreased caregivers’ self-assessed burden. However, this study’s delayed intervention design did not make it possible to determine if the caregiver-inclusive intervention was superior to customary care, and it addressed only a single problematic activity that was identified by the dyad (e.g., difficulty bathing).

Given the substantial contribution of family caregiving to enhancing the functioning of individuals with disabilities, the potentially negative impacts of caregiver burden, and the limitations of previous research, the present study was conducted to examine the effects of a caregiver-inclusive, 6-week, experimental AT intervention on both AT user and caregiver outcomes compared with outcomes for a customary care group. The study had two primary hypotheses:

- **Hypothesis 1:** Following the intervention, family caregivers in the experimental, caregiver-inclusive group will report significantly fewer physical and psychological demands associated with dyad-identified activities than caregivers in the customary care group. Additionally, family caregivers in the experimental group will experience a significantly greater decrease in overall perceived burden than caregivers in the customary care group.
- **Hypothesis 2:** Following the intervention, AT users in the experimental group will report more functional autonomy than users in the customary care group.

## Methods

The protocol for this multi-site, mixed-methods RCT employed a single-blind, experimentally controlled design in which dyads consisting of care recipients and their family caregivers were randomly assigned to either the experimental, caregiver-inclusive group or the customary-care comparison group [20].

### Ethical approval and consent to participate

Written informed consent was obtained from all dyad members. The study was approved by the research ethics boards at each site in accordance with the Declaration of the World Medical Association: Institut Universitaire de Gériatrie de Montréal, West Island Health and Social Services Center, University of Ottawa, Bruyère Continuing Care, University of British Columbia – Providence Health Care Research Institute, and Simon Fraser University. The trial was registered at https://clinicaltrials.gov/ (Identifier: NCT01640470).

### Participants

To be included in the study, each dyad needed to contain a care recipient and that individual’s family care provider. Care recipients needed to be aged ≥55 years, live at home, have a mobility limitation, be referred for AT-related homecare services, and receive ≥4 h per week of unpaid assistance from a single individual with daily activities or social participation for at least one month. Care recipients were excluded if they had cognitive impairments that prevented them from completing study questionnaires. Family caregivers were eligible to participate if they provided ≥4 h of assistance per week to the care recipient.

The study occurred in the residences of participants living in the Lower Mainland of British Columbia, the Montreal Metropolitan area in Quebec, and the Greater Ottawa area in Ontario, Canada. Subjects were recruited in Montreal through the West Island Health and Social Services Center; in Vancouver through Vancouver Community Health Care Units; and in Ottawa through the Champlain Community Care Access Center. Recruitment occurred from April 2012 to January 2015. This length of recruitment was needed given the challenges associated with enrolling research dyads generally, and our exclusion of AT users with cognitive impairments, who represent a large proportion of home care clients [13]. As previously reported [21], assistive technology was funded differently in each province in the study. In general, smaller equipment (e.g., reachers and canes) would normally be purchased by the user in Ontario and British Columbia; whereas this equipment was sometimes available through Home Care in Quebec. Funding was generally available for 80% of the cost of larger equipment in Ontario and all of the cost in Quebec, although there were long wait-lists for services. In British Columbia very little public funding was available for equipment.

**Fig. 1:**
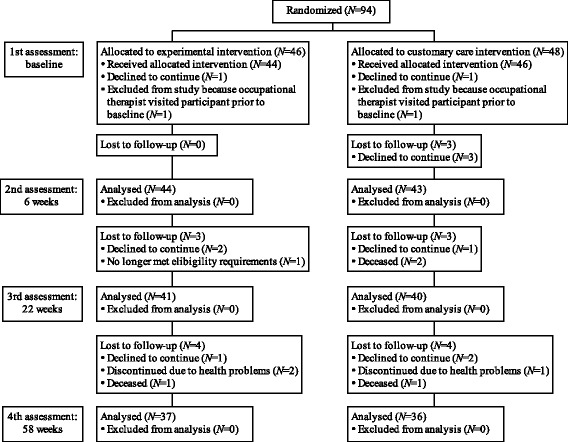
CONSORT flow diagram

Figure 1 presents a Consolidated Standards of Reporting Trials (CONSORT) diagram beginning with the randomization of participants. The enrollment information is not included in the diagram because the recruitment of dyads occurred through multiple third parties, and as a consequence the enrollment records for the Ottawa and Vancouver sites were not always supplied to the research coordinators. However, the enrollment records for the Montreal site indicate that 65 dyads were screened for eligibility and 45 were randomized to either the experimental group (*N* = 23) or the comparison group (*N* = 22). Randomization of dyads was conducted by research coordinators in blocks of 10 using online randomization software (https://www.random.org).

### Interventions

After randomizing the dyads, baseline data were collected, followed by delivery of the interventions. The experimental and comparison groups received interventions from different occupational therapists. The home-based Assistive Technology Provision, Updating, and Tune-Up (ATPUT) experimental intervention consisted of five components:

1. Working collaboratively with the care recipient and family caregiver, problematic activities were identified and prioritized;
2. The care recipient’s daily activities and social participation were assessed in the home and community;
3. Human assistance and AT being used at the time were reviewed;
4. The therapist made recommendations for changes in assistance and AT;
5. An ATPUT Personal Plan was negotiated by the occupational therapist with the care recipient and caregiver. This could include recommendations for AT, financial assistance to repair or acquire new AT, receipt of AT in a prompt manner, training, and additional follow-up visits.

The experimental intervention was delivered by registered occupational therapists who were trained by the first author to use an identical approach based on a treatment protocol containing 20 discrete steps [19]. Therapists in the experimental group recorded the interventions they provided and completed a checklist to indicate which steps in the protocol had been completed.

Participants assigned to the comparison group received customary care from the occupational therapist assigned to them by their center. This included the intervention normally provided to clients by the local health authorities. Because a standardized protocol was not used, family caregivers were not required to be included in the intervention process. Participants were provided with equipment based on local funding policies and received follow-up visits at the discretion of the therapists.

### Outcome measures

Five outcome measures were chosen for care recipients (AT users) and five for family caregivers. All measures were administered in the participants’ preferred language, French or English.

#### AT users

The primary outcome measure for AT users was a composite score of 13 items assessing mobility and performance with activities of daily living (ADL). The items were drawn from two subscales of the revised version of the Functional Autonomy Measurement System (SMAF) [22]. Total composite scores could vary between 0 (complete independence) and − 39 (complete dependence). A third subscale of the SMAF measuring performance with instrumental activities of daily living (IADL) was used as a secondary outcome measure. The IADL subscale comprises eight items rated with the same response scale used by the mobility and ADL subscales. The communication and mental functioning subscales of the SMAF were not used as they were unlikely to be affected by the interventions. The revised version of the overall SMAF illustrated good test-retest (Intraclass Correlation Coefficient [ICC] = .95) and inter-rater reliability (ICC = .96) when applied to a general population of adults age 65 years or older who presented with a significant loss of independence [22].

The Self-Reported Functional Independence Measure (SR-FIM) [23] and the Reintegration to Normal Living Index (RNLI) [24] were used as secondary outcome measures for AT users. The AT users’ levels of functional independence on tasks relating to self-care, sphincter control, transfers, and locomotion were reported by both AT users themselves (SR-FIM_U_) and caregivers (SR-FIM_CG_). Each of the 13 items comprising the four SR-FIM subscales were rated on a 7-point response scale, with 7 indicating complete independence and 1 indicating total dependence. The RNLI was used to determine how AT users managed activities, roles, and relationships. The 11-item, 10-point Likert-type version of the original scale was used. It had excellent internal consistency (Cronbach *α* = .91) when applied to participants living in the community with chronic conditions, e.g., spinal cord injury, stroke, or cerebral palsy [25].

#### Family caregivers

The primary outcome measure for family caregivers was the Caregiver Assistive Technology Outcomes Measure (CATOM) [19, 26]. Items 1-14 identify specific activities for which the caregiver provides assistance to the care recipient, subsequently assessing the frequency of assistance and the perceived psychological and physical burden associated with it. The activity-specific section includes questions about verbal cuing, physical assistance, caregiver pain, and worry about the possibility of care recipient or caregiver injury. Each item is rated on a 5-point scale, with higher composite scores indicating less pain and worry. The original CATOM was developed to assess single problematic activities, whereas the current study used a revised version which enabled dyads to identify the three problematic activities of highest priority. In the original study, the internal consistency of the activity-specific section was *α* = .73 [19].

The overall burden section of the CATOM, items 15-18, was used as a secondary outcome measure. It is comprised of four items rated on the same 5-point response scale. The internal consistency of this section was *α* = .78 [19]. Another secondary outcome measure for assessing caregiver burden was the composite score of three subscales from the Caregiver Burden Inventory (CBI) [27]. The items assess feelings of burden due to restrictions on caregivers’ time, missing out in life because of caregiving duties, and physical burden (Cronbach *α* range, .85-.86). Each of the 14 items is scored from 0 to 4, with higher cumulative scores indicating greater feelings of burden. Finally, caregiver health status was measured using the European Quality of Life descriptive system (EQ-5D DS) and visual analog scale (EQ-5D VAS) [28]. Each of the 5 items comprising the EQ-5D DS is scored from 1 to 3, with higher total scores indicating worse health. The EQ-5D VAS is scored on a scale of 0-100, where 100 is the best imaginable state of health.

### Sociodemographic and clinical variables

Sociodemographic data were collected from all study participants, including age, sex, ethnic origin, level of education, language, marital status, relationship between members of the dyad, as well as care-recipients’ duration of functional problems, diagnoses, and amount of formal caregiving received, if any. The cognitive status of care recipients was measured with the Montreal Cognitive Assessment (MoCA), a cognitive screening test designed to be sensitive to mild deficits [29]. It has good test-retest reliability (correlation coefficient = .92) and internal consistency (Cronbach *α* = .83). Adherence with the intervention was assessed by asking AT users and their caregivers how frequently they followed therapists’ AT recommendations and what proportion of recommended modifications they made to their environments. Responses were provided on a scale of 0-10, with higher scores indicating better adherence. Participants were also asked whether any problems were encountered while following therapists’ recommendations.

Information relating to the receipt and cost of AT was obtained using the Life Changes Form, a questionnaire created for that and other purposes. Caregivers’ attendance and/or awareness of intervention recommendations were derived from chart reviews and occupational therapists’ experimental intervention documentation.

### Data collection

With the exception of the SMAF, data were collected separately from care recipients and caregivers. The data collectors were blinded regarding participants’ intervention group. Sociodemographic and outcome data were collected at baseline (Time 0), and follow-up data were collected at week 6 (Time 1), week 22 (Time 2), and week 58 (Time 3). The primary outcome measures were administered first to dyad members, followed by the secondary measures in random order.

### Quantitative data analysis

#### Sociodemographic characteristics and outcome variables

Sociodemographic characteristics as well as the health-related and outcome variables were assessed for normal distribution using the 1-sample Kolmogorov-Smirnov test and visual inspection of histograms. Continuous variables were expressed as means, and categorical variables as proportions. Baseline differences between the experimental and comparison groups were compared using *t-*tests or Mann Whitney *U* tests for continuous data and *χ*^2^ for nominal data. Statistical analyses were performed using IBM SPSS Statistics 23. Values of *P* < .05 were considered to be statistically significant.

For primary and secondary outcome variables, mixed-effects models were used to control for repeated measurements that included baseline and 6-, 22-, and 58-week follow-up data. Time and intervention conditions were treated as fixed factors, and their interactions determined. Initial investigations revealed no fixed effects for site, and so the variable was not included in the models. Significant main effects for time were assessed using Sidak pairwise comparisons and expressed using mean difference scores and 95% confidence intervals (CI).

Covariation between changes in AT users’ primary outcome for functional autonomy (SMAF ADL and mobility subscales) and caregivers’ activity-specific perceived burden (CATOM items 1-14) for the dyad-identified problematic activities were assessed using bivariate Pearson Correlation.

#### Interventions

Mann Whitney *U* tests were used to compare the experimental and comparison groups in terms of the number of AT devices provided for problematic activities, the percentage of problematic activities targeted with AT, the cost of AT, the delay receiving AT, adherence with intervention recommendations, and the number of visits made by occupational therapists. To quantify treatment fidelity in the experimental group, the percentage of completed steps in the treatment protocol was calculated.

### Qualitative data analysis

Qualitative interviews were conducted with eight occupational therapists at the end of the study to document their experience of delivering interventions. The interviews were digitally recorded, transcribed verbatim, checked for accuracy, and analysed thematically [30]. Transcripts were reviewed multiple times to develop a sense of the data and carry out preliminary coding. The transcripts and preliminary codes were reviewed several additional times and further sub-coding was conducted. These codes and sub-codes were then categorized into themes. This analysis was facilitated using qualitative data analysis software (NVivo 10).

## Results

### Baseline

Baseline sociodemographic characteristics and outcome data for the care recipients and caregivers are presented in Tables 1 and 2, respectively. Only one significant difference was observed between the groups. Participants receiving the experimental intervention were more likely to report receiving formal care services, although this may have been a statistical artifact given the larger number of comparisons. Care recipients were approximately 75 years old and most were married. Family caregivers were approximately 65 years old and were mostly female spouses.

**Table 1:** Care recipients’ background characteristics and outcome measure scores at baseline

	Experimental Group (*N =* 44) mean ± SD or *N* (%)	Comparison Group (*N =* 46) mean ± SD or *N* (%)	*P*
Recipients’ background characteristics (range)			
Age (Yrs)	74.5 ± 10.4	75.4 ± 10.7	.688
Sex (Female)	25 (56.8)	24 (52.2)	.678
Ethnic origin (Canadian)	27 (61.4)	31 (67.4)	.661
Language of use (English)	36 (81.8)	39 (84.8)	.782
Years of education	13.3 ± 3.6	12.8 ± 4.2	.518
Marital status			.761
Married/common-law	30 (68.2)	36 (78.2)	
Unmarried/separated/divorced/widowed	14 (31.8)	10 (21.7)	
Primary diagnoses			.963
Osteoarthritis	15 (34.1)	17 (37.0)	
Cardiorespiratory	3 (6.8)	3 (6.5)	
Neurological	18 (40.9)	16 (34.8)	
Other	8 (18.2)	10 (21.7)	
Duration of functional problems (Yrs)	6.3 ± 8.2	4.3 ± 7.3	.100
Paid caregiving services (Yes)	19 (42.2)	11 (23.9)	.048
Months receiving paid care	30.6 ± 50.0	12.3 ± 27.3	.128
Hours/week paid care received	2.1 ± 2.4	3.0 ± 4.8	.794
MoCA (0-30)	23.0 ± 4.7	23.1 ± 4.7	.979
Primary outcome			
SMAF ADL & mobility sub-scales total (−39 - 0)	−8.8 ± 4.8	− 8.1 ± 4.4	.464
Secondary outcomes			
SMAF IADL sub-scale (−24 - 0)	−11.6 ± 4.0	−12.2 ± 4.4	.503
SR-FIM_U_ (13-91)	74.4 ± 14.2	75.2 ± 12.6	.786
SR-FIM_CG_ (13-91)	71.3 ± 12.8	72.0 ± 12.6	.787
RNLI (0-110)	75.1 ± 20.3	67.6 ± 18.5	.066

*Abbreviations: ADL* activity of daily living, *IADL* instrumental activity of daily living, *MoCA* Montreal Cognitive Assessment, *RNLI* Reintegration to Normal Living Index, *SMAF* Functional Autonomy Measurement System, *SR-FIM*_*U/CG*_ self-report Functional Independence Measure (user, caregiver)

**Table 2:** Caregivers’ background characteristics and outcome measure scores at baseline

	Experimental Group (*N =* 44) mean ± SD or *N* (%)	Comparison Group (*N =* 46) mean ± SD or *N* (%)	*P*
Caregiver background characteristics (range)			
Age (Yrs)	62.8 ± 12.5	67.4 ± 14.3	.109
Sex (Female)	25 (56.8)	29 (64.4)	.461
Language of use (English)	33 (76.7)	37 (80.4)	.797
Live with user (Yes)	37 (84.1)	39 (84.8)	.928
Years of education	14.3 ± 2.7	13.2 ± 3.4	.108
Employed (Yes)	12 (27.9)	13 (28.3)	.970
Relationship with user			.390
Spouse	27 (61.4)	35 (76.1)	
Child	15 (34.1)	10 (21.7)	
Extended kin or non-kin	2 (4.5)	1 (2.2)	
Years helping user	7.3 ± 7.1	6.6 ± 11.2	.169
Average hours/week providing assistance	29.2 ± 35.8	22.0 ± 23.9	.506
Average # of activities assisted with	9.7 ± 3.4	10.0 ± 3.0	.667
Caregiver primary outcome			
CATOM items 1-14 (14-70)	47.1 ± 10.8	49.7 ± 10.2	.236
Caregiver secondary outcomes			
CATOM items 15-18 (4-20)	14.5 ± 4.1	15.1 ± 4.3	.492
CBI (0-56)	20.5 ± 11.6	19.9 ± 12.4	.811
EQ-5D descriptive items (5-15)	6.6 ± 1.5	6.6 ± 1.2	.862
EQ-5D perceived health (0-100)	73.7 ± 19.4	78.9 ± 12.7	.139

Data are presented as mean ± SD or *n* (%)

*Abbreviations: CATOM* Caregiver Assistive Technology Outcome Measurement, *CBI* Caregiver Burden Inventory, *EQ-5D* European Quality of Life index

Problematic activities identified by the recipient-caregiver dyads included transferring (*n* = 24, 26%), negotiating stairs (*n* = 13, 14%), walking inside (*n* = 16, 17.5%), outdoor mobility (*n* = 12, 13%) and bathing (*n* = 12, 13%). Caregivers also reported providing assistance with instrumental activities including housekeeping (*n =* 76, 84%), meal preparation (*n* = 74, 81%), shopping (*n* = 78, 86%), laundry (*n* = 68, 75%), and transportation (*n* = 66, 73%).

### Interventions

The experimental group received more AT devices ($\overline{\mathrm{x}}$ = 1.73) to assist with problematic activities compared to the comparison group ($\overline{\mathrm{x}}$ = .98 devices; *P* = .01). Moreover, the experimental group had a significantly higher percentage of problematic activities targeted by the provision of AT ($\overline{\mathrm{x}}$ = 46.6%) compared to the comparison groups ($\overline{\mathrm{x}}$ = 26.3%; *P* = .006). The overall cost of AT did not differ significantly between the experimental groups ($\overline{\mathrm{x}}$ = 39.3CAD) and the comparison groups ($\overline{\mathrm{x}}$ = 43.7CAD; *P* = .386), nor did the time awaiting delivery of the AT (experimental group $\overline{\mathrm{x}}$ = 3.02 weeks, comparison group $\overline{\mathrm{x}}$ = 2.29 weeks; *P* = .345). Reports of adherence did not differ significantly between the groups. For example, the groups did not differ significantly in the proportion using the AT provided by the interventions (experimental group $\overline{\mathrm{x}}$ = 92.4%, comparison group $\overline{\mathrm{x}}$ = 72.1%; *P* = .153), nor did they differ significantly in the proportion implementing the recommended environmental modifications (experimental group $\overline{\mathrm{x}}$ = 75.5%, comparison group $\overline{\mathrm{x}}$ = 51.8%; *P* = .278). Occupational therapists providing the experimental intervention made significantly more visits to participants’ homes than did therapists providing the comparison group intervention (experimental group $\overline{\mathrm{x}}$ = 4.29 visits, comparison group $\overline{\mathrm{x}}$ = 2.79 visits; *P* = .001). Of relevance to treatment fidelity, an average of 93% of the steps comprising the experimental intervention were completed by occupational therapists providing that intervention.

#### Caregiver involvement

Caregivers’ involvement during therapist visits were determined from chart reviews and therapists’ experimental intervention documentation. In the experimental group, 75% of the caregivers were present during all therapist visits; the remainder missed one or two follow-up visits. Except for four caregivers, all were actively involved in identifying treatment goals. In the comparison group, 43% of caregivers were present during therapist visits. The same percentage (43%) were aware of the therapists’ visits and recommendations, but were not present during the visits. The remaining 13.5% neither attended the therapists’ visits nor evidenced awareness of their AT recommendations.

The qualitative interviews of the occupational therapists pointed to similarities between the interventions in terms of caregiver involvement, but subtle differences were spoken about as well. For example, therapists from both intervention groups commented on the importance of including caregivers in the intervention process and making them feel heard. Two of the four therapists who worked with the experimental group noted they more intentionally incorporated caregivers in the experimental intervention than was their customary practice. In the comparison group, two out of four therapists indicated they almost always incorporated the caregiver in their regular practice. However, they indicated that the degree of doing so depended on the recommended AT and/or the cognitive functioning of the care recipients.

### Primary and secondary outcomes

Results of the mixed-effects models for primary and secondary outcomes are presented in Table 3. Following the interventions, the functional autonomy of AT users in the experimental and comparison groups did not differ significantly (SMAF ADL & mobility, SMAF IADL, SR-FIM_U/CG_), and the groups did not differ significantly in their management of activities, roles, and relationships (RNLI). In both groups AT users showed significant declines over time in daily activity- and mobility-related functional autonomy (SMAF ADL & mobility), specifically between weeks 6 and 58 (mean difference = 1.16, 95% CI = .08-2.23) and between weeks 22 and 58 (mean difference = 1.34, 95% CI = .26-2.43). Additionally, there was a significant effect of time for RNLI scores for AT users in both groups, with a significant improvement between baseline and 22 weeks (mean difference = 5.65, 95% CI = .20-11.1).

**Table 3:** Mixed-effects models on outcome measures by group, time, and group by time interaction

Construct (range)	Group	Baseline, Mean (95% CI)	6 Weeks, Mean (95% CI)	22 Weeks, Mean (95% CI)	58 Weeks, Mean (95% CI)	Group, *F* (*P*)	Group × Time, *F* (*P*)	Time, *F* (*P*)
AT user primary outcome								
SMAF ADL & Mobility (−39 - 0)	E	−8.8 -(10.3-7.3)	−8.6 -(10.1-7.1)	−9.0 -(10.5-7.5)	−10.5 -(12.0-9.0)	1.90 (.171)	1.81 (.146)	**4.19 (.007)**
	C	−8.1 -(9.6-6.7)	−7.9 -(9.4-6.5)	−7.2 -(8.7-5.7)	−8.4 -(9.9-6.9)			
Secondary outcomes								
SMAF IADL (−24 - 0)	E	−11.6 -(12.9-10.3)	−11.7 -(13.0-10.4)	−12.2 -(13.6-10.9)	−12.8 -(14.1-11.4)	.054 (.817)	**4.66 (.004)**	.140 (.936)
	C	−12.2 -(13.4-10.9)	−12.5 -(13.8-11.2)	−11.8 -(13.1-10.5)	−11.1 -(12.4-9.7)			
SR-FIM_U_ (13-91)	E	74.4 (70.5-78.2)	72.3 (68.5-76.2)	72.5 (68.7-76.4)	71.7 (67.8-75.6)	1.77 (.187)	1.86 (.137)	.830 (.479)
	C	75.2 (71.5-78.9)	75.8 (72.0-79.6)	77.5 (73.6-81.3)	75.7 (71.8-79.6)			
SR-FIM_CG_ (13-91)	E	71.3 (67.5-75.2)	70.2 (66.3-74.1)	70.7 (66.8-74.6)	69.7 (65.7-73.7)	1.47 (.229)	1.39 (.246)	1.05 (.373)
	C	72.1 (68.3-75.9)	74.2 (70.4-78.1)	75.1 (71.2-79.0)	72.6 (68.6-76.6)			
RNLI (0-110)	E	75.1 (69.5-80.7)	78.4 (72.7-84.2)	76.3 (70.5-82.2)	76.1 (70.2-82.1)	1.35 (.249)	1.98 (.118)	**2.81 (.040)**
	C	67.5 (61.9-73.0)	71.9 (66.0-77.7)	77.7 (71.8-83.5)	73.7 (67.7-79.8)			
Caregiver primary outcome								
CATOM 1-14 (14-70)	E	46.9 (43.6-50.1)	48.8 (45.5-52.1)	50.5 (47.2-53.8)	50.0 (46.7-53.4)	2.69 (.105)	.278 (.841)	**7.12 (.001)**
	C	49.8 (46.6-53.0)	52.1 (48.8-55.3)	53.3 (50.0-56.6)	54.4 (51.1-57.8)			
Secondary outcomes								
CATOM 15-18 (4-20)	E	14.5 (13.2-15.7)	15.1 (13.8-16.4)	15.6 (14.2-16.9)	15.6 (14.3-17.0)	1.35 (.249)	.275 (.844)	**4.48 (.004)**
	C	15.1 (13.8-16.3)	16.4 (15.1-17.7)	16.3 (14.9-17.6)	16.7 (15.3-18.0)			
CBI (0-56)	E	20.5 (17.0-24.0)	20.3 (16.8-23.8)	19.6 (16.0-23.2)	20.3 (16.7-24.0)	.894 (.347)	.652 (.582)	1.38 (.250)
	C	19.9 (16.4-23.3)	18.1 (14.6-21.6)	17.0 (13.4-20.6)	17.3 (13.6-20.9)			
EQ-5D descriptive items (5-15)	E	6.6 (6.2-7.0)	6.9 (6.5-7.4)	6.8 (6.4-7.2)	6.9 (6.5-7.4)	.873 (.353)	.829 (.479)	1.25 (.293)
	C	6.6 (6.2-7.0)	6.5 (6.1-6.9)	6.5 (6.1-6.9)	6.7 (6.3-7.1)			
EQ-5D perceived health (0-100)	E	73.7 (69.0-78.3)	75.1 (70.3-79.8)	75.5 (70.7-80.3)	75.1 (70.1-80.0)	1.52 (.222)	.514 (.673)	.414 (.743)
	C	78.9 (74.3-83.4)	77.3 (72.6-82.0)	79.8 (74.9-84.7)	76.5 (71.5-81.5)			

*Abbreviations: ADL* Activities of daily living, *IADL* Instrumental activities of daily living, *CATOM* Caregiver Assistive Technology Outcome Measurement, *CBI* Caregiver Burden Inventory, *EQ-5D* European Quality of Life index, *RNLI* Reintegration to Normal Living Index, *SMAF* Functional Autonomy Measurement System, *SR-FIM*_*U/CG*_ Self-Reported Functional Independence Measure (user/caregiver)

Bold numbers: significant time, group, or time X group interaction effects

Family caregivers in the experimental group did not show significant reductions in their frequency of perceived physical and psychological burden associated with problematic activities compared to the comparison group (CATOM items 1-14), and overall caregiver burden and health were not significantly different between groups (CATOM items 15-18, CBI, EQ-5D). However, there was a significant main effect of time for activity-specific and overall burden, with both groups reporting significant reductions in burden scores between baseline and 22 weeks (CATOM 1-14: mean difference = 3.60, 95% CI = 1.13-6.06; CATOM 15-18: mean difference = 1.16, 95% CI = .07-2.26), and between baseline and 58 weeks (CATOM 1-14: mean difference = 3.88, 95% CI = 1.35-6.40; CATOM 15-18: mean difference = 1.39, 95% CI = .28-2.50).

Between baseline and 6 weeks, changes in AT users’ functional autonomy (SMAF ADL & mobility) were significantly correlated with changes in caregivers’ activity-specific burden (CATOM 1-14) for the experimental group (*r* = .513, *P* = .001), but not the comparison group (*r* = .040, *P* = .809). Similarly, between baseline and 22 weeks, the two scores were significantly correlated for the experimental group (*r* = .525, *P* = .001), but not the comparison group (*r* = .319, *P* = .062). Finally, between baseline and 58 weeks, the two scores were moderately correlated for the comparison group (*r =* .347, *P* = .051), but were less so for the experimental group (*r* = .236, *P* = .173).

#### Problematic activities

The primary and secondary outcome measures yielding the findings described in Table 3 did not distinguish among each dyad’s three highest priority problematic activities identified by use of the CATOM. To assess possible impacts of the interventions on the separate problematic activities, each was categorized according to the SMAF item applicable to it (e.g., difficulty with bathing was grouped with the SMAF item, “Washing”). Exploratory mixed-effects models were fitted to determine potential main effects for activities involving washing, toileting, transferring, and using the stairs. Compared with the customary care group, the caregiver-intensive group did not exhibit significantly improved outcomes for washing (*F*[1,53] = .670, *P* = .417), toileting (*F*[1,42] = 3.14, *P* = .084), transferring (*F*[1,33] = 1.56, *P* = .221), or using the stairs (*F* [1,9]=.020, *P* = .890).

## Discussion

This study was designed to test the hypothesis that an experimental, caregiver-inclusive approach to AT provision produces better outcomes for both care recipients and caregivers than does the approach that characterizes customary care. Contrary to that hypothesis, the experimental and customary-care comparison groups did not differ significantly with respect to AT-user functional autonomy or caregiver burden at any time point following the interventions. Across both groups over time, however, AT user functional autonomy decreased whereas caregiver burden was reduced. These findings ensued even though the experimental intervention targeted more problematic activities, provided more AT to participants, and included more visits from occupational therapists.

There are two main possible explanations for the lack of differences between the groups post-intervention. The first explanation is that, as implemented, the experimental, caregiver-inclusive intervention differed less from the customary care condition than was expected. As a result of the unintended caregiver involvement with customary care, treatment differentiation likely was not achieved. The intent had been to have caregivers in the experimental group much more strongly engaged in the process of AT provision than their counterparts in the comparison group. However, evidence to support that expectation did not materialize. Contrary to the assumption that customary care would not necessarily involve caregivers, therapists for the customary care group indicated they often engaged caregivers in their regular practice. They may have done so likely knowing the experimental intervention emphasized caregiver engagement. The net result would have been blurring of differences between the interventions as actually carried out. Intended differences between experimental and customary care interventions may have been attenuated as well by having attracted caregivers to the overall study who, by virtue of the informed consent process, supported its focus on caregiving and who were predisposed to participate actively in interventions. Supporting this idea, admission guidelines for the present study required that caregivers were providing twice as many hours of assistance per week (at least four hours rather than two) as required by the previous, delayed-intervention study [19].

With previous efficacy studies, better treatment differentiation was achieved between the experimental and control groups [7–9]. The experimental interventions for some RCTs involved AT provision and assistance with problem solving strategies for one year or more [7, 9], whereas the control groups received no such assistance through the studies [7–9]. As a result, control group participants’ dependence on community-available health care services and on appointments with physicians resulted in them obtaining significantly less AT [7, 8], or no AT at all [9]. Similarly, a delayed-intervention study which compared participants who received an AT intervention with those who had not yet received one also found significant differences in outcomes [19].

A second plausible explanation for the lack of evidence pointing to differences between the impacts of the experimental and customary care interventions is possible insensitivity of the outcome measures. This question about instrument sensitivity applies to both the care-recipient outcome measures and the caregiver ones. The primary outcome measures may not have been sensitive to the multifaceted nature of the interventions, which targeted a variety of specific problematic activities. For example, the SMAF covered 13 different aspects of ADLs or mobility, but both interventions may have addressed only a few of these; even a single one in some cases. With the CATOM, the problematic activities were collectively evaluated by each item. Therefore, the primary outcome measures for both AT users and caregivers may have been unable to detect significant changes in particular problematic activities, or alternatively, to reveal that some activities improved while others declined.

In addition, it is possible that the SMAF was insufficiently responsive compared with the Assessment of Life Habits, the primary measure used in the previous delayed intervention study [19]. The Assessment of Life Habits captures users’ feelings of satisfaction and their self-reported accomplishment performing activities, whereas the primary (SMAF) and secondary (SR-FIM) outcomes assessed in the present study measure self-reported or observed independence in performing activities. It is possible that small changes in AT users’ self-reported or observed independence performing activities might not have been captured by the measures in the present study, but might have produced significant changes in satisfaction.

AT user outcomes did not demonstrate a universal pattern of decline among primary and secondary outcomes. Mann et al. [7] and Wilson et al. [8] observed overall declines in function, similar to the primary outcome measure (SMAF) in the present study. The time related decline of functional status may reflect a general diminution of functional capability and/or performance for older adults with disabilities [8]. Despite declines in function, AT users in both groups reported significant improvements with their management of activities, roles, and relationships (RNLI). It may be that both interventions had a direct impact on the RNLI because it had items that evaluated AT users’ feelings of satisfaction, similar to the Assessment of Life Habits used in the delayed-intervention study [19]. Although the satisfaction measured by the RNLI and Assessment of Life Habits applies to different actions (i.e., social participation vs. performing activities), the improvements noted in both studies highlights the importance of measuring outcomes for constructs other than functional performance.

The finding that decreased activity-specific burden for caregivers was positively correlated with increased functional autonomy for AT users at the 6-week follow-up was similar to findings from the delayed-intervention study [19], even though it employed a different outcome measure for AT users. The correlation between AT users’ and caregivers’ outcomes remained significant at 22 weeks, but it declined by 58 weeks, possibly reflecting the worsening of AT users’ functional performance between 22 and 58 weeks.

The caregiver outcomes did not vary consistently between primary and secondary outcome measures. Activity-specific and overall caregiver burden (measured using the CATOM) decreased significantly over time, but the CBI did not reveal significant decreases in burden, although both groups’ scores trended toward it. A potential explanation is that the CBI was developed for caregivers of those with cognitive impairments and included items about unpredictable behaviors and complete loss of ability to perform daily activities, which were likely less relevant to participants in our study. In any case, if there was an unintended congruence between experimental and customary care interventions in terms of caregiver involvement, this suggests that caregiver-inclusive interventions may positively impact caregivers across a variety of different interventions.

It is possible that the decline in caregiving burden is attributable to a general adaptation process involving how effectively the demands of caregiving are addressed. The adaptation may be both psychological (e.g., resulting in greater self-efficacy) and behavioral (e.g., resulting in greater efficiency). Because AT was a key component of the interventions for both groups, this study’s finding of decreased burden may also reflect previous suggestions that provision of AT can reduce caregiver burden [18, 19]. Considering that longitudinal data show general increases in burden over time [31], the latter interpretation would provide support for the potential benefits of assistive technology which could be facilitated through programs that enable access to devices, prescription services, and necessary follow up.

### Limitations

Two main limitations of the study should be noted. The adherence questionnaire evaluated general usage of AT and environmental modification completed at each follow-up time point, but it did not explore adherence with each intervention specifically. Another potential limitation relates to this study’s inability to control for the intervention provided through customary care. This study assumed that the interventions would be different, but the frequent inclusion of caregivers in the customary care group likely blurred the distinction between the interventions and obscured potentially different outcomes. As a result, a larger sample size would likely have been necessary to detect differences between the interventions.

### Future directions

Given the apparent similarities between the caregiver-inclusive and customary care interventions, a better way to test this study’s hypotheses might have been to include a third arm in which caregivers were excluded. Unfortunately it is difficult to justify excluding caregiver involvement. So perhaps an alternative might be to use a quasi-experimental design to compare outcomes from AT users who receive substantial assistance from caregivers and AT users who have very little caregiver involvement. Rather than focusing exclusively on the primary family caregiver, future research could also explore the impact of AT on all of the caregivers involved (e.g., formal caregivers, and multiple informal caregivers (e.g., family, friends, neighbours)).

In the present study, significant differences between the groups may have been found if different constructs had been measured. For example, enhancing safety is one of several reasons why therapists may recommend specific AT devices [32], but safety is not among the factors expressly evaluated by the SMAF and is only assessed with three items on the CATOM. Future studies may benefit from measuring safety, whether self-assessed or observer-assessed, that is applicable to a variety of functions, e.g., ambulation, transferring, and bathing, and thus to a variety of AT devices, e.g., walkers, lifts, and grab bars.

Finally, there are potential ways for future studies to increase the intensity of the experimental intervention for both AT users and caregivers. As in previous RCTs [7–9], the duration and intensity of the interventions can be increased with monthly telephone or video follow-ups to determine details of the usage of the provisioned AT and provide assistance related to problematic activities or use of AT. The scope of the intervention could also be increased to allow for IADLs to be targeted, as was done by Gitlin et al. [9] Given that family caregivers provided a substantial amount of assistance with IADLs, targeting instrumental activities would likely lead to further reductions in burden.

## Conclusions

A single-blind, mixed-methods, randomized controlled trial was conducted to investigate whether a six-week long caregiver-inclusive assistive technology intervention would improve care recipients’ functional independence and decrease caregivers’ sense of burden. Although the experimental intervention addressed significantly more dyad-identified problematic activities, similar caregiver involvement was evident in both groups. This may explain why there was no significant differences in outcomes between the groups over time. However, despite significant declines in functional autonomy, caregivers’ activity-specific and overall burden decreased significantly in both groups. This suggests that there may be beneficial effects to assistive technology provision, which involves caregivers in that process. Further research is needed to confirm these study findings.

## Abbreviations

ADL: Activities of daily living
AT: Assistive technology
ATPUT: Assistive Technology Provision, Updating, and Tune-Up
CATOM: Caregiver Assistive Technology Outcomes Measure
CBI: Caregiver Burden Inventory
CI: Confidence intervals
CONSORT: Consolidated Standards of Reporting Trials
EQ-5D DS: European Quality of Life descriptive system
EQ-5D VAS: European Quality of Life visual analog scale
IADL: Instrumental activities of daily living
ICC: Intraclass correlation coefficient
MoCA: Montreal Cognitive Assessment
RCT: Randomized controlled trial
RNLI: Reintegration to Normal Living Index
SMAF: Functional Autonomy Measurement System
SR-FIM: Self-report Functional Independence Measure
